# *Laetiporus sulphureus* polysaccharides mitigate colitis by reshaping the gut microbiota and regulating immune responses

**DOI:** 10.3389/fphar.2026.1705032

**Published:** 2026-04-15

**Authors:** Sharafat Ali, Yamina Alioui, Imran khan, Hidayat Ullah, Mujeeb Ur Rahman, Aamna Atta, Mohammed Abusidu, Muhammad Ilyas, Uzma Noor, Renzhen Ma, Muhsin Ali, Nabeel Ahmed Farooqui, Ting Deng, Guangyang Wang, Yi Xin, Shanshan Sha, Yufang Ma

**Affiliations:** 1 Department of Biochemistry and Molecular Biology, College of Basic Medical Science, Dalian Medical University, Dalian, China; 2 Department of Biotechnology, College of Basic Medical Science, Dalian Medical University, Dalian, China; 3 Department of Microecology, College of Basic Medical Science, Dalian Medical University, Dalian, China; 4 Guangdong Provincial Key Laboratory of Research and Development of Natural Drugs, and School of Pharmacy, Guangdong Medical University, Dongguan, China; 5 Department of Physiology, College of Basic Medical Sciences, Dalian Medical University, Dalian, China

**Keywords:** epithelial barrier, gut microbiota, immune modulation, *Laetiporus sulphureus* polysaccharides, ulcerative colitis

## Abstract

**Background:**

Inflammatory bowel disease (IBD) involves epithelial barrier disruption, immune dysregulation, and microbial imbalance. The present study investigated the protective mechanisms of *Laetiporus sulphureus* polysaccharides (LSP) in dextran sulfate sodium (DSS)-induced colitis, focusing on intestinal barrier restoration, immunomodulation, and gut microbiota remodeling.

**Methods:**

LSP was structurally characterized using HPLC, FTIR, and SEM analyses, revealing a heteropolysaccharide primarily composed of glucose (55.16%), galactose (16.55%), and mannose (13.52%). Experimental colitis was induced in BALB/c mice with 3% DSS, followed by oral LSP administration (200 or 400 mg/kg). Disease severity, histopathology, barrier markers, cytokine profiles, macrophage polarization, and gut microbiota composition were evaluated using biochemical assays, immunofluorescence, IHC, and 16S rRNA sequencing.

**Results:**

LSP significantly mitigated DSS-induced colitis by reducing the disease activity index by approximately 60% (∼2.5-fold, p < 0.001) and restoring colon length (∼1.5-fold, p < 0.01). Barrier integrity improved *via* enhanced mucin-2 expression (∼3.5-fold) and tight junction proteins Occludin, Claudin-1, and ZO-1 (∼5–9-fold). LSP suppressed pro-inflammatory cytokines TNF-α, IL-6, and IL-1β (∼2–3-fold) while upregulating anti-inflammatory mediators IL-10 and TGF-β (∼2.5–3-fold), reflecting a rebalanced mucosal immune milieu. 16S rRNA sequencing demonstrated reversal of DSS-induced dysbiosis, characterized by a reduction in pathogenic Escherichia–Shigella (∼3.8-fold) and Enterobacteriaceae (∼3.5-fold), and enrichment of beneficial taxa including *Lactobacillus*, Bifidobacterium, and Ruminococcus (∼2–4-fold).

**Conclusion:**

LSP exerts multi-targeted protection against colitis by reinforcing epithelial barrier function, attenuating inflammation, and reshaping gut microbial ecology. These findings highlight LSP as a promising natural therapeutic candidate for IBD. Further metabolomic and meta transcriptomic analyses are warranted to elucidate the microbial metabolites and molecular pathways mediating these protective effects.

## Graphical Abstract

**Figure:**
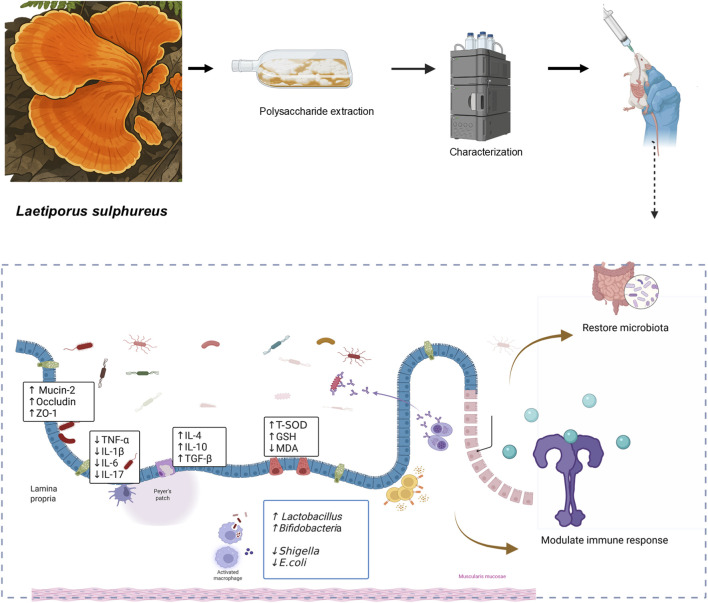
Schematic representation of *Laetiporus sulphureus* polysaccharides (LSP) in DSS-induced colitis. LSP treatment restores epithelial barrier integrity, attenuates oxidative stress, rebalances mucosal immunity, and reshapes gut microbiota, collectively alleviating colitis pathology. https://BioRender.com.

## 1 Introduction

The gastrointestinal tract plays a pivotal role in maintaining human health, integrating nutrient absorption, immune surveillance and host microbe interactions (Sanz et al., 2025; Armet et al., 2022; Shang et al., 2024). Inflammatory bowel disease (IBD) comprising Crohn’s disease (CD) and ulcerative colitis (UC) is a chronic relapsing disorder of this system with rising global prevalence and a substantial burden on patient health and healthcare systems (Buie et al., 2023; Diez-Martin et al., 2024; Sanmarco et al., 2022). IBD is characterized by recurrent intestinal inflammation impaired epithelial barrier function, and dysregulated immune responses. Despite significant advances in understanding its immunopathology the precise etiology remains incompletely defined reflecting a complex interplay of genetic susceptibility, environmental exposures, epithelial dysfunction and aberrant mucosal immunity (Xie et al., 2024; Jarmakiewicz-Czaja et al., 2022). Among these factors intestinal microbial dysbiosis, a state of altered microbial diversity and functionality, has emerged as a central determinant influencing both disease onset and progression (Jergens et al., 2021; Gasaly et al., 2025; Barbara et al., 2021). The pathogenesis of ulcerative colitis (UC) is primarily associated with an imbalance in immune regulation marked by excessive production of pro-inflammatory cytokines such as IL-17, IL-23, IL-6, TNF-α and IL-1β. This aberrant cytokine profile perpetuates chronic colonic inflammation and compromises epithelial barrier integrity (Neurath, 2014). Activated immune cells, particularly macrophages and T-helper subsets further exacerbate the inflammatory cascade. Conversely, the expression of anti-inflammatory mediators, including TGF-β and IL-10 is often reduced in UC, undermining immune homeostasis and impairing mucosal healing processes (Maloy and Powrie, 2011). Moreover disruption of tight junction proteins such as ZO-1, Occludin, and Claudin-1 leads to impaired epithelial barrier function, increased intestinal permeability and subsequent microbial translocation into the mucosa (Peterson and Artis, 2014).

Current pharmacological approaches including amino salicylates, corticosteroids, immunosuppressants and biologics primarily aim to induce remission by controlling inflammation (Elhag et al., 2022). However long-term use is constrained by adverse effects, high costs and diminishing efficacy. Corticosteroid dependence can lead to severe complications such as osteoporosis metabolic syndrome and immunosuppression (Chotiyarnwong and McCloskey, 2020) while nonsteroidal anti-inflammatory drugs exacerbate gastrointestinal injury and are generally contraindicated in IBD (Tai and McAlindon, 2021). These limitations have intensified the search for safer multi-targeted therapies capable of simultaneously modulating immune responses and microbial composition. Such challenges have directed attention toward natural compounds particularly polysaccharides which offer multi-faceted therapeutic potential (Guo et al., 2024).

Natural polysaccharides have attracted considerable attention as therapeutic candidates owing to their biocompatibility, low toxicity and broad pharmacological activities including antioxidant, anti-inflammatory, immunomodulatory and prebiotic effects (Bai et al., 2022; Tian et al., 2023; Mwangi et al., 2022). Present in fungi plants and marine organisms these macromolecules can simultaneously modulate multiple pathways implicated in IBD pathogenesis. Medicinal mushrooms in particular, are rich sources of bioactive polysaccharides (Durairajan and Thangaraj, 2024). Mushroom-derived polysaccharides from *Ganoderma lucidum, Lentinula edodes*, and *Morchella esculenta* have shown protective effects in colitis models through antioxidant activity, immune regulation, and modulation of the gut microbiota (Kurl et al., 2025). For example, *G. lucidum* polysaccharides restored microbial diversity and suppressed colonic inflammation in DSS-induced colitis (Li L. et al., 2022; Qiao et al., 2025), while *L. edodes* extracts enhanced barrier integrity and increased SCFA-producing taxa. Similarly, *M. esculenta* polysaccharides alleviated colitis by downregulating pro-inflammatory cytokines and promoting beneficial commensals (Li M. et al., 2022).

However, despite the growing evidence for mushroom polysaccharides in gut health, the effects of *Laetiporus sulphureus*, a widely distributed medicinal fungus, remain unexplored.

Among these *Laetiporus sulphureus* emerges as a particularly promising candidate combining traditional medicinal use with a rich repertoire of bioactive compounds.

*Laetiporus sulphureus* an edible basidiomycete widely distributed across Asia, Europe and North America has long been used in traditional medicine to treat gastrointestinal and inflammatory disorders (Patocka, 2019; Adamska, 2023). Phytochemical analysis reveal that it contains a diverse array of bioactive compounds including polysaccharides, triterpenoids, phenolic acids and lectins many of which exhibit anti-inflammatory antitumor and hepatoprotective activities (Yadav and Negi, 2021; Duan et al., 2022; Jovanović et al., 2023). Despite these promising properties, the therapeutic potential of *L. sulphureus*-derived polysaccharides (LSP) in intestinal inflammation has not been systematically explored and mechanistic studies in experimental colitis remain limited.

To address this gap, we investigated the protective effects of crude LSP in a murine model of dextran sulfate sodium (DSS)-induced colitis. We hypothesized that LSP could mitigate colitis by modulating inflammatory responses, preserving epithelial barrier integrity and restoring gut microbial homeostasis. To test this hypothesis, we employed a comprehensive experimental framework combining histopathological evaluation, analyses of tight junction and mucin proteins, profiling of cytokines and oxidative stress markers and 16S rRNA-based gut microbiota characterization. This integrative approach provides mechanistic insights into the multi-targeted therapeutic potential of LSP for IBD.

## 2 Materials and methods

### 2.1 Chemicals and reagents

Fruiting bodies of *L. sulphureus* were obtained from Shandong Tai’an Yinsheng Food Co., Ltd. (Shandong, China). Dextran sulfate sodium (DSS) was purchased from Yeasen Biotechnology (Shanghai, China). Protein concentrations were determined using a bicinchoninic acid (BCA) assay kit (Jiancheng Bioengineering Institute, Nanjing, China). Enzyme-linked immunosorbent assay (ELISA) kits for TNF-α, IL-1β, IL-6, IL-17, IL-4, and IL-10 were procured from Jiangsu Meibiao Biotechnology Co., Ltd. (China). Primary antibodies against zonula occludens-1 (ZO-1), occludin, claudin-1, and mucin-2 (MUC2) were supplied by Proteintech Group (Wuhan, China). Horseradish peroxidase (HRP)-conjugated secondary antibodies and a 3,3′-diaminobenzidine (DAB) chromogen detection system were obtained from ZSGB-BIO (Beijing, China). All chemicals and reagents were of analytical grade and purchased from standard commercial suppliers.

### 2.2 Extraction of polysaccharides from *L. sulphureus* (LSP)

Crude polysaccharides were isolated from *L. sulphureus* fruiting bodies by following a slightly modified procedure based on an earlier report (Su et al., 2017) to obtain water soluble fractions for subsequent biochemical analyses. Dried fruiting body powder was suspended in deionized water at a 1:30 (w/v) ratio and subjected to hot water extraction at 85 °C for 3 h to solubilize polysaccharides. The extract was filtered concentrated under reduced pressure and precipitated by adding four volumes of chilled 95% ethanol. The suspension was incubated at 4 °C overnight to ensure complete precipitation. The precipitate was collected by centrifugation at 2,000 × g for 15 min, redissolved in deionized water, and treated with α-amylase (2 mg/mL, 60 U/mL) and α-glucosidase (1 mg/mL, 50 U/mL) to remove residual starch. To further purify the polysaccharides, the solution was dialyzed (12–14 kDa molecular weight cut-off) against deionized water at 4 °C for 72 h, with water replaced every 8 h. Proteins were removed by treatment with 1.5% (v/v) trichloroacetic acid (TCA). The resulting solution was freeze-dried to yield crude *L. sulphureus* polysaccharides (LSP), which were used for downstream experiments. While these steps reduce impurities, trace proteins, phenolics, or other non-polysaccharide components may remain. Future work will focus on further purification using column chromatography or other advanced separation techniques to isolate the bioactive fractions.

### 2.3 Chemical composition and structural analysis of LSP

The chemical composition and structural features of crude LSP were analyzed to determine carbohydrate content, monosaccharide composition, functional groups and surface morphology. Total carbohydrate content was quantified using the phenol sulfuric acid method following a standard colorimetric protocol. Monosaccharide composition was assessed by high-performance liquid chromatography (HPLC) according to established methods enabling determination of the sugar constituents of LSP (Zhao et al., 2023). Functional groups were characterized by Fourier Transform Infrared (FTIR) spectroscopy. LSP was mixed with potassium bromide at a 1:100 (w/w) ratio, pressed into transparent disks, and analyzed using a Shimadzu FTIR-4200 spectrometer (Shimadzu, Japan). Spectra were recorded over 500–4,000 cm^−1^ with a resolution of 4 cm^−1^ and 10 scans per second, allowing identification of characteristic polysaccharide bonds.

Surface morphology and microstructural features were examined by scanning electron microscopy (SEM). Dried polysaccharide powder was mounted on self-adhesive carbon tape sputter coated with gold and imaged using a HITACHI S-2600N SEM (Hitachi, Tokyo, Japan) at an acceleration voltage of 15 kV.

### 2.4 Animal husbandry and study design

Animal experiments were conducted to evaluate the biological effects of LSP under controlled conditions. Male BALB/c mice (n = 32, 4–5 weeks old) were purchased from Liaoning Changsheng Biotechnology Co., Ltd. (Liaoning, China). Upon arrival animals were acclimated for 1 week in a specific pathogen-free (SPF) facility. Housing conditions were maintained at 22 °C ± 3 °C with 50% ± 5% relative humidity under a 12 h light/dark cycle. Mice had *ad libitum* access to distilled water and a standard SPF diet (Shenyang Mao Hua Biotechnology Co., Ltd., Shenyang, China).

### 2.5 Ethical approval and animal care

All animal experiments were conducted in accordance with institutional and national guidelines for the care and use of laboratory animals, and the protocol was approved by the Animal Care and Research Ethics Committee of Dalian Medical University (approval number: 202310265). Mice were monitored twice daily for body weight, stool consistency, and rectal bleeding. Humane endpoints were predefined: animals losing more than 20% of body weight, exhibiting severe lethargy, or developing rectal prolapse were immediately euthanized. To minimize pain and distress, buprenorphine (0.05 mg/kg, s. c.) was administered when signs of discomfort (e.g., hunching, piloerection, reduced activity) were observed. All efforts were made to reduce animal suffering and to use the minimum number of animals required to achieve statistical validity. Mice were euthanized *via* intraperitoneal injection of sodium pentobarbital (200 mg/kg; 54 mg/mL working solution from a 325 mg/mL stock; injection volume 70–110 μL based on body weight 18–30 g), as described by (Rahman et al., 2025). Death was confirmed by cervical dislocation following cessation of respiration. All procedures conformed to institutional regulations and the AVMA Guidelines for the Euthanasia of Animals (Underwood and Anthony, 2020).

### 2.6 DSS-induced colitis and LSP administration

A dextran sulfate sodium (DSS)-induced murine colitis model was established to evaluate the protective effects of LSP. Following a 1-week acclimatization period mice were randomly assigned to a normal control group (NC, n = 8) or a DSS-exposed group (n = 24). Colitis was induced in the DSS group by administering 3% DSS (molecular weight 36,000–50,000 Da) in drinking water for 7 days while NC mice received water alone. From day 8 DSS-exposed mice were returned to standard drinking water and subdivided into three cohorts (n = 8 each): DSS (no LSP treatment), low-dose LSP (200 mg/kg) and high-dose LSP (400 mg/kg). LSP treatments were administered orally once daily for 15 consecutive days. NC and DSS groups received equivalent volumes of phosphate-buffered saline (PBS). These doses were selected based on preliminary dose-ranging experiments (100–800 mg/kg) which demonstrated that doses below 100 mg/kg produced minimal protective effects, while higher doses (800 mg/kg) offered no additional benefit and were therefore excluded from the final design. The selected doses were effective and well tolerated, with no signs of acute toxicity in DSS-induced colitis mice.

**FIGURE 1:**
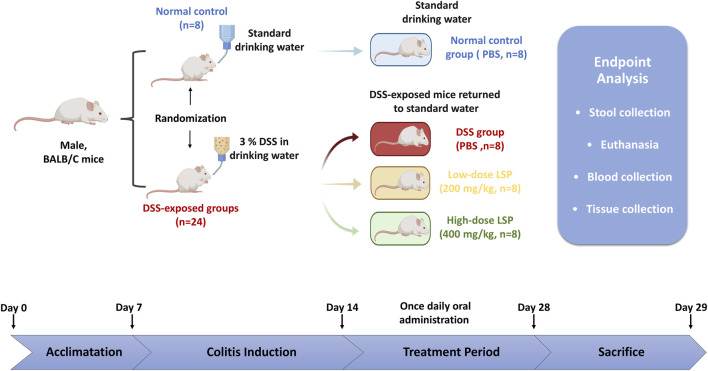
Experimental design and treatment plan: Colitis was induced in mice by administering 3% DSS in drinking water for 7 days. The colitis mice were divided into 3 groups (n = 8): no LSP treatment (DSS); low-dose 200 mg/kg LSP (LSP_L); and high-dose 400 mg/kg LSP (LSP_H). A separate control group received sterile water instead of DSS (NC). The treatment was administered for 15 days following colitis induction. Stool samples were collected, and mice were sacrificed on day 29 for analysis.

On day 28 all mice were euthanized under anesthesia and blood samples were collected for serum analyses. Figure 1 depicts the experimental design and treatment plan, including group allocation, DSS induction protocol, LSP dosing regimen, and the 29-day study timeline.

### 2.7 Assessment of colitis severity and organ indices

Disease severity in DSS-treated mice was assessed using a modified Disease Activity Index (DAI) protocol (Calvete-Torre et al., 2024) incorporating body weight loss, stool consistency and rectal bleeding. Body weight loss was scored from 0 to 4 (0, none; 1, 1%–5%; 2, 5%–10%; 3, 10%–20%; 4, >20%). Stool consistency was graded as 0 (normal) 1 (soft) or 4 (diarrhea), and rectal bleeding was scored as 0 (absent) 2 (occult) or 4 (gross). The sum of these scores was recorded as the DAI for each mouse. Body weight was measured daily, and food and water intake were recorded every 3 days to monitor general health status. At the study endpoint mice were euthanized by cervical dislocation and colons were excised, cleaned and weighed. Organ indices were calculated as the ratio of organ weight (mg) to body weight (g) to evaluate systemic and immunological responses to DSS exposure and LSP treatment.

### 2.8 RT–qPCR and ELISA for gene expression and cytokine quantification

Molecular and immunological responses to DSS-induced colitis and LSP treatment were assessed by analyzing gene expression and cytokine production in colon tissue.

Total RNA was extracted from colon samples using Trizol reagent (Thermo Fisher Scientific United States) and stored at −80 °C. Complementary DNA (cDNA) was synthesized by reverse transcription and quantitative real-time PCR (RT–qPCR) was conducted using a SYBR Green RT–qPCR kit (Takara, Japan) and ChamQ Universal SYBR qPCR Master Mix (Vazyme, China) on a Bioer LightGene 9600 system (Hitech, China). Expression of inflammatory and regulatory genes including IL-1β, IL-4, IL-17, IL-10, TNF-α, IL-23, FOXP3, GATA3, and TGF-β was quantified with β-actin serving as the reference gene. Primer sequences are provided in Supplementary Table S1. All reactions were performed in triplicate. Relative expression levels were calculated using Gene 9600 software and analyzed in GraphPad Prism 10 (GraphPad Software, United States). Cytokine levels in colon tissue were determined by ELISA to validate transcriptional findings. Colon tissues were homogenized in PBS, centrifuged at 3,500 rpm for 20 min at 4 °C and supernatants were stored at −80 °C. Concentrations of IL-4, TNF-α, IL-6, IL-10 and IL-1β were quantified using commercial ELISA kits (Meibiao Biotechnology Co., Ltd., China) according to the manufacturer’s instructions.

### 2.9 Evaluation of neutrophil infiltration and oxidative stress in colitis

Inflammatory cell infiltration and oxidative stress in colon tissue were evaluated to assess the local and systemic effects of LSP on DSS-induced colitis.

Neutrophil infiltration was quantified by measuring activity of myeloperoxidase (MPO), a marker of neutrophils macrophages and monocytes. Colon tissues were homogenized and MPO was determined using a commercial kit (Cusabio Technology, China) according to the manufacturer’s instructions. MPO activity served as an indicator of local inflammation and neutrophil infiltration.

Oxidative stress was assessed by measuring T-SOD, catalase (CAT), malondialdehyde (MDA) and GSH levels in colon tissue. Tissue homogenates were prepared and centrifuged following the same protocol as for MPO analysis. Protein concentrations in the supernatants were determined using a bicinchoninic acid (BCA) assay (Jiancheng Bioengineering Institute, Nanjing, China) to normalize enzyme and biomarker activities. Systemic oxidative stress and inflammatory status were further evaluated by measuring serum nitric oxide (NO) concentrations using a commercial assay kit according to the manufacturer’s instructions.

### 2.10 Histological assessment of colonic tissue

Structural and inflammatory changes in the colon were assessed to evaluate DSS-induced injury and the effects of LSP treatment. Distal colon segments were excised post-euthanasia and fixed in 4% paraformaldehyde at room temperature for 24 h to preserve tissue architecture. Fixed tissues were embedded in paraffin, and 3 µm-thick sections were prepared using a microtome. Sections were deparaffinized in xylene, rehydrated through a graded ethanol series, and stained with hematoxylin and eosin (H&E) for histological analysis. Histopathological evaluation was performed using a Leica microscope (Leica Microsystems, Wetzlar, Germany). A semi-quantitative scoring system was applied to assess inflammation and epithelial regeneration. Inflammation was graded from 0 to 3 (0 absent; 1 slight; 2 moderate; 3 severe). Epithelial regeneration was scored from 0 to 4 (0 complete regeneration or normal architecture; 1 near-complete regeneration; 2 regeneration with crypt depletion; 3 loss of surface epithelium; 4 complete absence of tissue repair). This scoring system allowed precise quantification of histological damage and the restorative effects of LSP on colonic architecture.

### 2.11 Evaluation of mucosal barrier integrity and goblet cell morphology

Mucosal barrier integrity and goblet cell morphology were assessed by immunohistochemical (IHC) and histochemical staining of colonic tissue.

Paraffin-embedded colon sections (5 µm) were mounted on positively charged slides, deparaffinized in xylene and rehydrated through a graded ethanol series. Antigen retrieval was performed prior to immunostaining using the SP-KIT9720 kit (MXB Biotechnologies, Beijing, China) according to the manufacturer’s protocol. Mucin-2 (MUC2) expression was quantified semi-quantitatively across three randomly selected fields per slide by a blinded investigator.

Goblet cell morphology and mucus layer integrity were evaluated using Periodic Acid–Schiff (PAS) staining. Sections were incubated with periodic acid for 5 min, treated with Schiff reagent for 10 min rinsed counterstained with hematoxylin, dehydrated through graded ethanol cleared in xylene and mounted with neutral balsam (Solarbio, Cat-G8590). Epithelial acidic mucins, including sulfated and non-sulfated forms, were differentiated using Alcian Blue (AB) staining at pH 2.5. After standard deparaffinization and hydration, sections were incubated with 1% aqueous alizarin blue acetate for 10 min oxidized with 1% periodic acid for 5 min treated with Schiff reagent for 10 min and processed through washing dehydration, clearing and mounting. All assessments were performed by an investigator blinded to experimental groups.

### 2.12 Assessment of intestinal barrier integrity and macrophage infiltration

Intestinal barrier integrity and colonic macrophage infiltration were evaluated *via* immunofluorescence. Paraffin-embedded colon sections (5 µm) were mounted on positively charged slides, deparaffinized, and rehydrated. Antigen retrieval was conducted in citrate buffer using a microwave (350 W, 15 min) followed by blocking with 5% bovine serum albumin (BSA) for 30 min at room temperature. Sections were incubated overnight at 4 °C with primary antibodies against ZO-1, Occludin, and Claudin-1 (Proteintech, Wuhan, China). After PBS washes, slides were incubated with FITC-conjugated goat anti-rabbit secondary antibody (Proteintech) for 1 h at room temperature. Nuclei were counterstained with DAPI for 5 min, and slides were imaged using a fluorescence microscope. Colonic macrophage infiltration and polarization were assessed on similarly processed sigmoid colon sections using primary antibodies against CD68 (1:500), CD86 (1:2,000), and CD163 (1:500; Proteintech). Sections were incubated with FITC-conjugated goat anti-rabbit IgG secondary antibody for 1 h at room temperature nuclei counterstained with DAPI and mounted with antifade medium. Fluorescence intensity and spatial distribution were quantified in a blinded manner using ImageJ software to evaluate macrophage density and polarization.

### 2.13 Fecal microbiota profiling to assess DSS-Induced colitis and LSP treatment effects

The impact of DSS-induced colitis and LSP treatment on intestinal microbial communities was assessed using 16S rRNA gene sequencing.

Fecal samples were aseptically collected, immediately flash-frozen in liquid nitrogen, and stored at −80 °C to preserve microbial integrity. Genomic DNA was extracted using a commercial kit (Qiagen, MD, United States) according to the manufacturer’s instructions. The V4 region of the bacterial 16S rRNA gene was amplified using universal primers 515F (5′-GTGCCAGCMGCCGCGGTAA-3′) and 806R (5′-GGACTACHVGGGTWTCTAAT-3′). Amplified products were purified, normalized, pooled, and prepared for sequencing libraries using the KAPA Library Quant Kit (KAPA Biosystems, Wilmington, MA, United States). High-throughput sequencing was performed on an Illumina HiSeq 4000 platform to generate 2 × 150 bp paired-end reads.

Raw sequencing reads underwent quality control, including removal of low-quality and chimeric sequences using the Chimera, Chime algorithm. Clean reads were clustered into operational taxonomic units (OTUs) at 97% sequence identity using Vsearch v1.11.1, and taxonomic assignments were performed against the SILVA128 database. Shared and unique OTUs among experimental groups were visualized using Venn diagrams to evaluate community overlap and distinctiveness. Alpha diversity metrics, including observed species, Chao1, ACE, Shannon, and Simpson indices, were calculated using QIIME to assess species richness and evenness within samples. Beta diversity, representing inter-group differences in microbial composition, was analyzed using Bray-Curtis dissimilarity matrices and visualized *via* principal coordinate analysis (PCoA), principal component analysis (PCA), and non-metric multidimensional scaling (NMDS). Functional potential of microbial communities was inferred using BugBase, and linear discriminant analysis effect size (LEfSe) was applied to identify taxa differentially abundant among treatment groups, with results presented as taxonomic cladograms.

### 2.14 Statistical analysis

All statistical analyses were conducted using GraphPad Prism version 10.5 (GraphPad Software, United States). Quantitative data are presented as mean ± standard deviation (SD) unless otherwise specified. Intergroup differences were evaluated using one-way analysis of variance (ANOVA) followed by Tukey’s *post hoc* test to account for multiple comparisons. The probability value of p < 0.05 was considered statistically significant. For microbiota analyses, 16S rRNA gene sequencing data were processed and interpreted using the QIIME and R software platforms. Differences in microbial community composition were assessed using the Kruskal–Wallis test, while Mann–Whitney U tests were applied to compare operational taxonomic unit (OTU) abundances and predicted phenotypic features. Functional and ecological inferences were derived using STAMP, FAPROTAX, and BugBase, enabling identification of statistically significant variations in taxonomic distribution, metabolic pathways, and microbial phenotypes across experimental groups. These integrated analytical approaches ensured methodological rigor, statistical robustness, and reproducibility in both experimental and bioinformatics evaluations.

## 3 Results

### 3.1 Structural characterization of *Laetiporus sulphureus* polysaccharides (LSP)

#### 3.1.1 Monosaccharide composition of LSP

**FIGURE 2:**
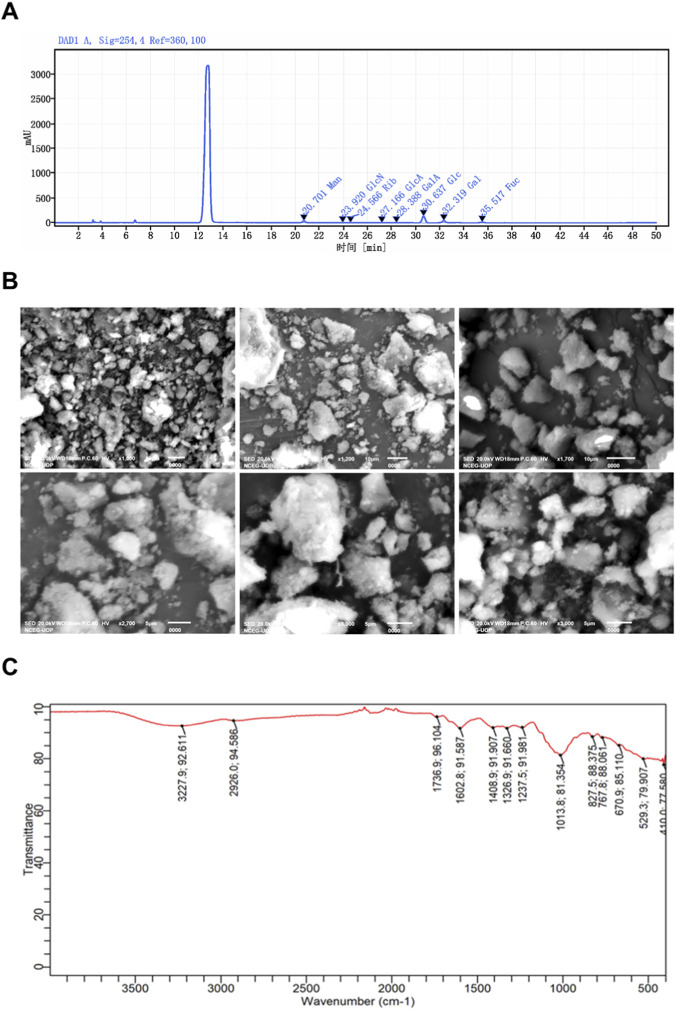
Characterization of *Laetiporus sulphureus* polysaccharides (LSP). **(A)** High-performance liquid chromatography (HPLC) analysis of LSP showing peak times corresponding to glucose (∼13 min), galactose, mannose, fucose, rhamnose, glucuronic acid, and galacturonic acid. **(B)** Scanning electron microscopy (SEM) images of LSP at magnifications of ×1,000, ×2,000, and ×3,000 illustrating surface morphology and granular structure. **(C)** Fourier-transform infrared spectroscopy (FTIR) spectrum of LSP displaying characteristic absorption peaks of functional groups.

All experiments were performed using a single, well-characterized batch of LSP to ensure compositional consistency. Total carbohydrate content, determined by the phenol–sulfuric acid assay, was 48.13 mg/mL, with an extraction yield of 19%. Residual protein content was low (1.3%), confirming effective enrichment of carbohydrate macromolecules and supporting its classification as a crude polysaccharide. Monosaccharide composition was analyzed by HPLC following acid hydrolysis (Figure 2A). LSP consisted predominantly of neutral sugars, including glucose (55.16%), galactose (16.55%), mannose (13.52%), fucose (12.63%), and rhamnose (1.16%). Acidic sugars were detected only in minor amounts, specifically glucuronic acid (0.59%) and galacturonic acid (0.39%). Thus, neutral sugars accounted for more than 98% of the detected monosaccharide composition, whereas uronic acids represented a minimal fraction. These results confirm that LSP is a carbohydrate-dominant heteropolysaccharide primarily composed of neutral monosaccharide residues with minor acidic components. Further structural analyses would be required to elucidate its detailed glycosidic architecture (Table 1).

**TABLE 1:** Composition of crude *Laetiporus sulphureus* polysaccharide.

Component	Concentration (mg/kg)	Percentage %
Mannose	103,525.33	13.5281488
Ribose	8,912	1.16457356
Glucuronic acid	4,362.67	0.5700909
Galacturonic acid	3,008	0.39069711
Glucose	422,117.33	55.1600854
Galactose	126,677.33	16.5535311
Fucose	96,656	12.6305007

#### 3.1.2 Morphological features of LSP

We investigated the microstructural organization of LSP to assess how particle morphology might influence solubility and biological interactions. SEM imaging at magnifications of ×1,000 to ×3,000 revealed irregular, compact and granular aggregates with rough and porous surfaces (Figure 2B). The particle sizes were heterogeneous with larger block-like structures interspersed among smaller granules indicative of variable aggregation and possible branching of the polysaccharide chains. The observed surface porosity and irregularity may enhance interaction with solvents and biological macromolecules suggesting that LSP morphology could contribute to its functional and bioactive properties. While these observations provide foundational morphological insight, advanced structural analyses are necessary to fully characterize polysaccharide architecture.

#### 3.1.3 Functional group analysis of LSP

To further elucidate LSP structural features we performed FTIR spectroscopy to identify characteristic chemical groups (Figure 2C). The spectrum displayed a broad O–H stretching band at 3,227 cm^−1^, indicative of extensive hydrogen bonding, and a C–H stretching peak at 2,926 cm^−1^. Signals at 1,763 cm^−1^ corresponded to uronic acids, while bands at 1,602 and 1,404 cm^-1^ reflected asymmetric and symmetric COO^−^ stretching. Absorptions between 1,236 and 1,013 cm^−1^ confirmed the presence of glycosidic C–O–C and C–O–H linkages, and additional peaks at 927 and 867 cm^−1^ indicated β-glycosidic linkages, with a minor α-glycosidic contribution at 814 cm^−1^. The skeletal vibrations between 670 and 520 cm^−1^ further supported a stable polysaccharide framework. LSP has a carbohydrate backbone with mixed α-/β-linkages and uronic acids, providing a basis for its bioactivity. NMR, methylation, and linkage analyses are needed to fully define bioactive motifs.

### 3.2 LSP mitigates DSS-Induced colitis by preserving colon structure and redox homeostasis

**FIGURE 3:**
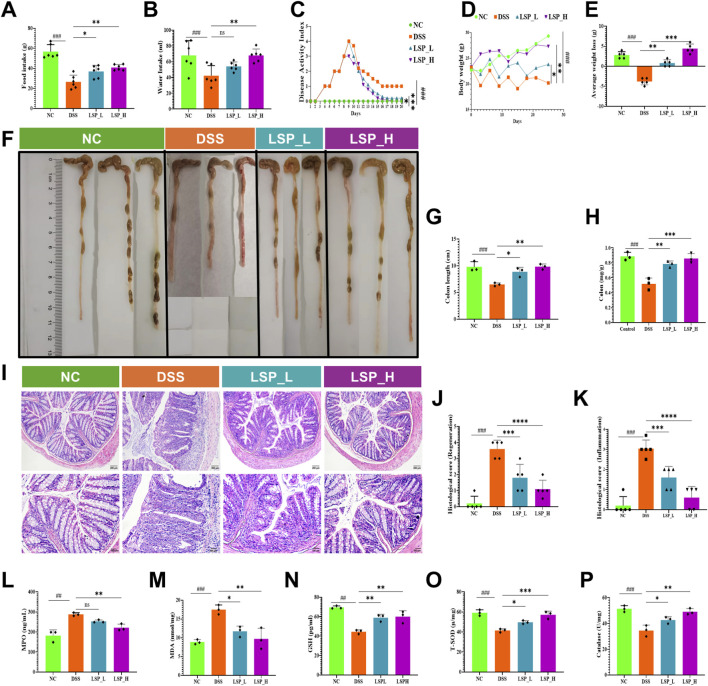
LSP attenuates DSS-induced colitis by improving disease parameters, histopathology, and redox balance. **(A,B)** Daily food and water intake; **(C)** Disease activity index (DAI); **(D,E)** Body weight and average weight loss; **(F)** Representative colon images; **(G,H)** Colon length and colon index; **(I)** Representative H&E-stained colon sections; **(J,K)** Histological regeneration and inflammation scores; **(L)** Myeloperoxidase (MPO) activity; **(M)** Malondialdehyde (MDA) levels; **(N–P)** Antioxidant markers: reduced glutathione (GSH), total superoxide dismutase (T-SOD), and catalase (CAT). All data were presented as mean ± SD. Statistical analysis was performed using one-way ANOVA followed by Tukey’s *post hoc* test. *p < 0.05, **p < 0.01, ***p < 0.001.

To evaluate whether LSP could protect against chemically induced colonic inflammation we investigated its effects on clinical histopathological and biochemical parameters in a DSS-induced murine colitis model (Figures 3A–P). DSS treatment significantly reduced food and water intake ∼2.1 and ∼1.6-fold (*p* < 0.001) respectively, compared to control group reflecting illness-induced anorexia and dehydration (Figures 3A,B). Administration of LSP restored these measures in a dose-dependent manner. Both low (LSP_L) and high (LSP_H) doses significantly improved food intake by ∼ 1.3 and ∼1.5-fold (*p* < 0.05 and *p* < 0.01) respectively, compared to DSS group (Figure 3A), whereas LSP_H recovered water consumption by ∼1.6-fold (*p* < 0.01) compared to DSS group (Figure 2B). Correspondingly, DSS markedly elevated the disease activity index (DAI) reflecting weight loss, diarrhea and bleeding (*p* < 0.001) which was dose-dependently attenuated by LSP (*p* < 0.05 for LSP_L and *p* < 0.001 for LSP_H) compared to DSS group (Figure 3C). Body weight and average weight loss were similarly improved in LSP treated mice (*p* < 0.05 and *p* < 0.001) respectively with stronger effects observed in the high-dose group (Figures 3D,E). Macroscopic examination revealed substantial DSS-induced colon shortening from ∼9.8 cm to ∼6.5 cm consistent with inflammation driven tissue contraction, which was partially reversed by both LSP treatments to ∼8.8 cm (*p* < 0.05) and 9.8 cm (*p* < 0.01) respectively (Figures 3F,G). Colon index measurements mirrored these findings where DSS reduced the index by ∼1.7 fold (*p* < 0.001). LSP doses attenuated the effect of DSS significantly, with LSP_L and LSP_H reversed the index by ∼1.5 (*p* < 0.01) and ∼1.7 (*p* < 0.001) respectively. LSP_H showed a more pronounced effect and recovered the index near to control values that indicates LSP alleviates the systemic and anatomical manifestations of DSS-induced colitis (Figure 3H). Histopathological analysis provided mechanistic insight into LSP protective effects. H&E staining revealed ∼18-fold (*p* < 0.001) increase in histological score indicating severe mucosal disruption and immune cell infiltration, and ∼15-fold increase in histological inflammatory score in DSS mice whereas LSP_L and LSP_H supplementation significantly improved epithelial regeneration by ∼2-fold and ∼3.6-fold, while preserved inflammation score by ∼1.8-fold and 5-fold (*p* < 0.01 and *p* < 0.001) respectively (Figures 3I–K). DSS exposure elevated MPO levels by ∼1.5-fold (*p* < 0.01) compared to control condition, LSP_H inhibited DSS effect and reduced neutrophil infiltration by ∼1.3-fold (*p* < 0.01) compared to DSS group, highlighting protective effect of LSP against acute inflammatory responses (Figure 3L).

Additionally, LSP modulated oxidative stress which is a central driver of colitis pathology. DSS treatment elevated MDA levels by ∼1.9-fold (*p* < 0.001) indicates lipid peroxidation, while these levels are significantly reduced by both LSP doses (*p* < 0.05 and *p* < 0.01) compared to DSS group (Figure 3M). Concurrently, the DSS-induced depletion of the GSH by ∼1.6-fold (*p* < 0.01) that was significantly restored by both LSP treatments by ∼1.3-fold (*p* < 0.01) compared to DSS group (Figure 3N). Key enzymatic defenses, including T-SOD and CAT were downregulated ∼1.4-fold and ∼1.5-fold (*p* < 0.001) respectively by DSS compared to control condition. LSP co-treatment enhanced the T-SOD levels by ∼1.1-fold (*p* < 0.05) and ∼1.4-fold (*p* < 0.01). Similarly, CAT levels recovered with LSP by ∼1.2-fold (*p* < 0.05) and ∼1.4-fold (*p* < 0.01) respectively, compared to DSS group (Figures 3O,P).

Together these findings demonstrate that LSP supplementation particularly at higher doses mitigates DSS-induced colitis by improving colon architecture, reducing inflammatory damage and restoring redox homeostasis.

### 3.3 Restoration of mucus secretion and tight junction proteins by LSP in DSS-induced colitis

**FIGURE 4:**
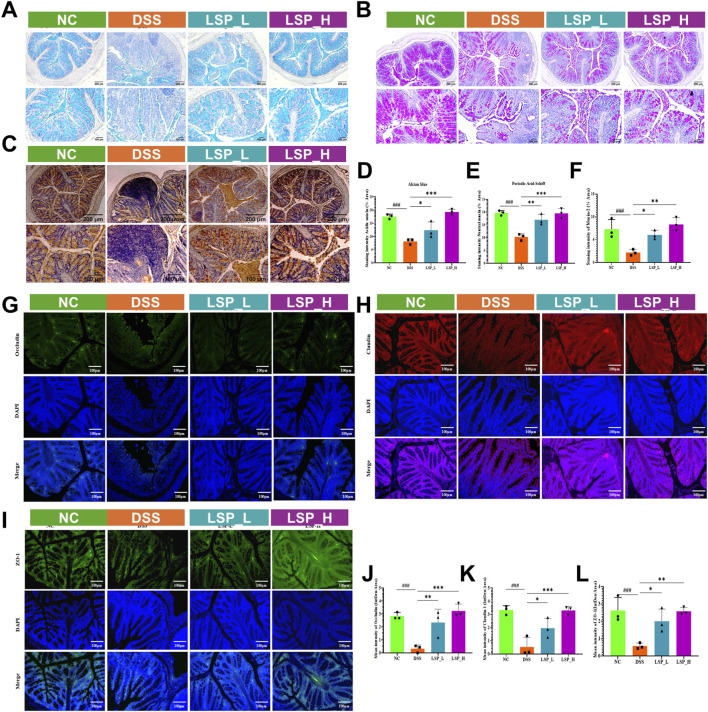
LSP improves mucosal barrier integrity in DSS-induced colitis. **(A–C)** Representative Alcian Blue (AB), Periodic Acid–Schiff (PAS), and immunohistochemical (IHC) staining of colonic sections; **(D–F)** Quantification of goblet cell markers from AB, PAS, and IHC; **(G–I)** Immunofluorescence (IF) images of tight junction proteins Occludin, Claudin-1, and ZO-1; **(J–L)** Quantification of IF signal intensities. LSP restored goblet cell staining and enhanced epithelial barrier protein expression in a dose-dependent manner. All data were presented as mean ± SD. Statistical analysis was performed using one-way ANOVA followed by Tukey’s *post hoc* test. *p < 0.05, **p < 0.01, ***p < 0.001.

To determine whether LSP could preserve epithelial barrier integrity and mucus production during colonic inflammation we assessed goblet cell function and tight junction protein expression in DSS-induced colitis (Figure 4). Alcian Blue (AB) and Periodic Acid–Schiff (PAS) staining revealed a pronounced loss of goblet cells and mucin content by ∼2.1-fold and ∼1.9-fold respectively (*p* < 0.001) in DSS-treated mice compared to control (Figures 4A,B). Both LSP_L and LSP_H significantly restored goblet cells and mucin, with LSP_H group showing near to control recovery in both compartments by ∼2.3-fold and ∼1.9-fold (*p* < 0.001) compared to DSS group (Figures 4D,E). IHC analysis further confirmed that DSS suppressed the mucin expression by ∼3.3-fold (*p* < 0.001). LSP treatments significantly improved mucin expression levels. LSP_H exerted stronger effects and enhanced the expression by ∼3.7-fold (*p* < 0.001) compared to DSS group (Figures 4C,F). These data indicate that LSP effectively rescues mucus-producing goblet cell function disrupted by DSS.

Next, tight junction protein expressions were examined to evaluate barrier integrity. IF results revealed that DSS exposure substantially reduced Occludin, Claudin-1 and ZO-1 levels by ∼9.3-fold, ∼6.6-fold, and ∼5.2-fold respectively, consistent with epithelial barrier disruption. Treatment with LSP_L and LSP_H significantly upregulated these proteins in a dose-dependent manner with LSP_H showing the strongest restoration across all markers where Occludin, Claudin-1, and ZO-1 expressions increased by ∼10.6-fold, ∼6.6, and ∼5-fold, respectively (Figures 4G–L).

Together, these findings demonstrate that LSP preserves epithelial barrier function during DSS-induced colitis by promoting goblet cell activity and maintaining tight junction protein expression highlighting its potential to protect the intestinal epithelium from inflammatory damage.

### 3.4 LSP modulates colonic cytokine networks to promote mucosal recovery

**FIGURE 5:**
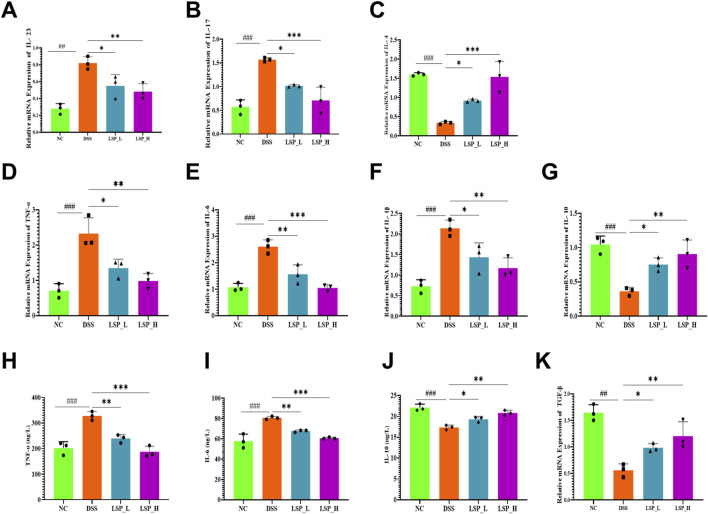
LSP regulates colonic cytokines in DSS-induced colitis. **(A–G)** mRNA levels of IL-23, IL-17, IL-4, TNF-α, IL-6, IL-1β, IL-10, and TGF-β; **(H–J)** protein levels of TNF-α, IL-6, IL-10; **(K)** TGF-β restoration. DSS increased pro-inflammatory and suppressed anti-inflammatory cytokines. LSP dose-dependently reversed these effects, with stronger responses at high dose. All data were presented as mean ± SD. Statistical analysis was performed using one-way ANOVA followed by Tukey’s *post hoc* test. *p < 0.05, **p < 0.01, ***p < 0.001.

Pro and anti-inflammatory cytokines were examined to see whether the structural improvements in mucosal integrity induced by LSP were accompanied by alterations in the local immune micro-environment (Figure 5). Furthermore, dysregulated cytokine signaling is a hallmark of colitis contributing to tissue injury and impaired healing, though therapeutic modulation of this network may promote mucosal recovery.

Our results showed that DSS exposure markedly increased the expression of the pro-inflammatory cytokines *IL-23* and *IL-17* by ∼3-fold (*p* < 0.01 and *p* < 0.001) compared to normal control. Both LSP_L and LSP_H significantly reduced these levels in a dose-dependent manner. LSP_L reduced the effect of DSS by ∼1.9-fold and 1.5-fold (*p* < 0.05 and *p* < 0.01) respectively, while LSP_H attenuated the expression by ∼1.6-fold and ∼2.1-fold (*p* < 0.05 and *p* < 0.001) respectively, indicating effective suppression of Th17-driven inflammatory pathways (Figures 5A,B). In contrast, DSS suppressed the anti-inflammatory cytokine *IL-4* by ∼5.3-fold (*p* < 0.001) which was significantly restored by both LSP treatments by ∼3-fold (*p* < 0.05) and ∼5-fold (*p* < 0.001) compared to DSS group (Figure 5C). Key pro-inflammatory mediators *TNF-*α*, IL-6,* and *IL-1*β were significantly elevated in DSS mice by ∼3.2-fold, ∼2.6-fold, and ∼3-fold (*p* < 0.001) respectively. LSP_L decreased these over-expressions by ∼1.6-fold, ∼1.7-fold, and ∼1.5-fold (*p* < 0.05–0.001) respectively, compared to DSS group. Similarly, LSP_H attenuated the levels by ∼2.5-fold, ∼2.6-fold, and ∼1.9-fold (*p* < 0.01–0.001) respectively, compared to DSS group (Figures 5D–F). Conversely *IL-10* was suppressed in DSS-treated mice (∼2.7-fold, *p* < 0.001) while ∼2.3-fold (*p* < 0.05) and ∼2.5-fold (*p* < 0.01) increased were noticed in LSP_L and LSP_H groups compare to DSS (Figure 5G).

ELISA results demonstrate that DSS exposure increased TNF-α and IL-6 protein-levels by ∼1.6-fold and ∼1.4-fold (*p* < 0.001), respectively. LSP_L decreased the levels of both proteins by ∼1.3-fold and ∼1.1-fold (*p* < 0.01) respectively, whereas LSP_H inhibited these levels by ∼1.7-fold and ∼1.3-fold (*p* < 0.001) respectively compared to DSS group (Figures 5H,I). Additionally, DSS reduced IL-10 expression by ∼1.3-fold (*p* < 0.001), while LSP_L and LSP_H recovered this expression by ∼1.1-fold (*p* < 0.05) and 1.2-fold (*p* < 0.01) compared to DSS group (Figure 5J). Subsequently, a critical mediator of mucosal healing, TGF-β was downregulated with DSS by ∼3.2-fold (*p* < 0.001) followed by reduction noticed in LSP_L and LSP_H by ∼1.7-fold (*p* < 0.05) and ∼2.4-fold (*p* < 0.01) respectively, compared to DSS group (Figure 5K). These results demonstrate that LSP not only facilitates structural recovery of the colonic mucosa but also orchestrates a shift in the mucosal immune milieu attenuating pro-inflammatory cytokine signaling while enhancing anti-inflammatory and reparative pathways. This coordinated immunomodulation likely contributes to the observed resolution of colitis and underscores the potential of LSP as a multifaceted therapeutic agent. While these data indicate robust immunomodulatory effects, the mechanistic basis whether direct interaction with immune cells or indirect *via* microbiota-derived metabolites cannot be determined from the current *in vivo* model.

### 3.5 LSP promotes anti-inflammatory macrophage polarization and regulatory T cell responses in colitis

**FIGURE 6:**
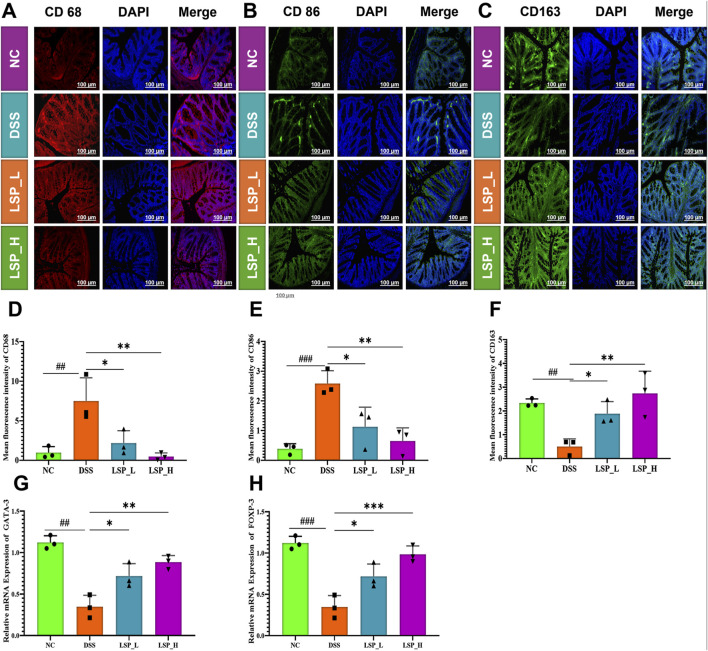
LSP modulates immune cell programs in DSS-induced colitis. **(A–C)** IF images of CD68, CD86, CD163 macrophages; **(D–F)** quantification showing DSS-induced macrophage infiltration and reversed by LSP; **(G,H)** GATA-3 and FOXP-3 expression in Th2 and Treg cells restored by LSP. All data were presented as mean ± SD. Statistical analysis was performed using one-way ANOVA followed by Tukey’s post hoc test. *p < 0.05, **p < 0.01, ***p < 0.001.

To elucidate the cellular mechanisms underlying LSP-mediated immunomodulation we examined whether LSP influences macrophage polarization and T cell transcriptional profiles in the colonic mucosa (Figure 6). Dysregulated macrophage activity and T cell differentiation are central drivers of colitis pathogenesis, whereas the modulation of immune homeostasis may promote tissue repair and inflammation resolution.

IF analysis revealed a pronounced accumulation of CD68 macrophages in DSS-treated mice indicating enhanced innate immune infiltration (Figures 6A–C). Quantitative assessment confirmed a significant increase in CD68 cells by ∼8.2-fold (*p* < 0.01) compared to control, which was substantially reduced following LSP_L and LSP_H administration by ∼3.5-fold (*p* < 0.05) and ∼16-fold (*p* < 0.01) respectively, compared to DSS monotherapy (Figure 6D). Markers of macrophage polarization further demonstrated that DSS induced a pro-inflammatory M1 phenotype evidenced by elevated CD86 expression by ∼6.7-fold (*p* < 0.01) while the anti-inflammatory M2 marker CD163 was suppressed ∼4.7-fold (*p* < 0.01). Both LSP_L and LSP_H restored this balance by decreasing CD86 by ∼2.2-fold (*p* < 0.05) and ∼4.1 (*p* < 0.01) while enhancing CD163 levels by ∼3.8-fold (*p* < 0.05) and ∼5.5-fold (*p* < 0.01) respectively, suggesting a shift toward an anti-inflammatory macrophage phenotype (Figures 6E,F).

Beyond innate immunity LSP also influenced key transcription factors governing adaptive T cell differentiation. DSS exposure suppressed GATA-3 (∼3.2-fold, *p* < 0.01) essential for Th2 lineage commitment and FOXP-3 (∼2.8-fold, *p* < 0.001) a critical regulator of regulatory T cells. LSP both treatments significantly restored the expression of both factors. LSP_L recovered the expressions of both GATA-3 and FOXP-3 by ∼2-fold (*p* < 0.05–0.01), while LSP_H restored these levels by ∼2.5-fold (*p* < 0.01) and ∼2.6-fold (*p* < 0.001) respectively, indicating a reprogramming of T cell responses toward anti-inflammatory and regulatory profiles (Figures 6G,H).

Together these results indicate that LSP modulates both innate and adaptive immune compartments in colitis promoting anti-inflammatory macrophage polarization and supporting regulatory T cell programs. This dual effect likely underpins the broad immunoregulatory and therapeutic actions of LSP in resolving intestinal inflammation. While these data demonstrate significant modulation of macrophage polarization and T cell regulatory profiles, the underlying mechanism whether LSP acts directly on immune cells or indirectly *via* microbiota-derived signals remains unresolved in the current *in vivo* model.

### 3.6 LSP reverses DSS-induced gut microbial dysbiosis and restores ecological diversity

**FIGURE 7:**
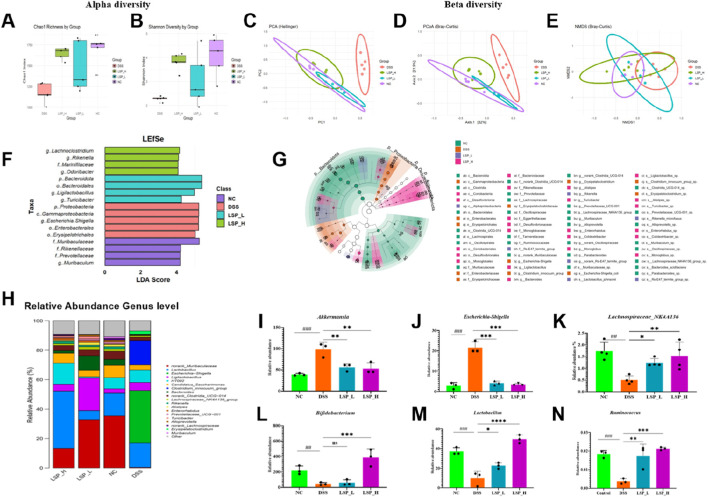
LSP restores gut microbial diversity and composition in DSS-induced colitis. **(A,B)** Alpha diversity indices (Chao1, Shannon) show DSS-induced loss of richness and evenness, restored by LSP in a dose-dependent manner. **(C–E)** Beta diversity analyses (PCA, PCoA, NMDS) reveal distinct microbial community structures, with LSP-treated groups shifting toward NC. **(F,G)** LEfSe analysis identifies differentially abundant taxa across groups. DSS enriches pro-inflammatory taxa (*Escherichia-Shigella,* Enterobacteriaceae*, Proteobacteria,* Erysipelotrichaceae), while LSP promotes beneficial genera (*Bacteroides*, Alistipes, Turicibacter, Ligilactobacillus for LSP_L; *Rikenella, Odoribacter, Lachnoclostridium, Marinifilaceae* for LSP_H). NC is dominated by *Muribaculaceae, Parabacteroides*, and *Alloprevotella.* **(H–N)** Genus-level comparisons: *Akkermansia and Escherichia-Shigella* elevated in DSS; LSP suppresses these while enriching *Bifidobacterium, Lactobacillus*, Lachnospiraceae*_NK4A136_group* and *Ruminococcus* (dose-dependent). All data were presented as mean ± SD. Statistical analysis was performed using one-way ANOVA followed by Tukey’s *post hoc* test. *p < 0.05, **p < 0.01, ***p < 0.001.

Given the immunological improvements observed in Figures 5, 6, we next investigated whether LSP administration influences gut microbial composition in DSS-induced colitis (Figures 7A–N).

To assess microbial richness and evenness alpha diversity indices were measured, alpha diversity analyses demonstrated that DSS significantly reduced microbial richness and evenness as reflected by lower Chao1 and Shannon indices compared to the NC group (Figures 7A,B) indicating loss of microbial complexity. LSP supplementation partially restored these indices at low dose (LSP_L; *p* < 0.05) and more robustly at high dose (LSP_H; *p* < 0.001) suggesting a dose-dependent recovery of ecological diversity. Similar trends were observed with ACE and Simpson indices (Supplementary Figure S1) further confirming that DSS-induced colitis disrupts microbial richness and evenness which LSP treatment can mitigate.

To further investigate group-specific differences in microbial community composition, beta diversity was assessed using multiple ordination methods. Principal component analysis (PCA) revealed clear separation along PC1 (48.42%) and PC2 (29.32%) (Figure 7C) while principal coordinate analysis (PCoA) demonstrated similar clustering patterns with PCoA1 and PCoA2 explaining 39.28% and 19.86% of the variation respectively (Figure 7D). Non-metric multidimensional scaling (NMDS) yielded a low stress value (0.05) confirming the reliability of the ordination (Figure 7E). Across all three approaches DSS-treated samples clustered distinctly from the NC group indicating a pronounced alteration in microbial structure. In contrast both LSP_L- and LSP_H-treated groups shifted closer to NC, with LSP_H exhibiting greater overlap suggesting a more substantial restoration of the microbial community.

Differential abundance analysis using LEfSe revealed microbial signatures associated with each group (Figures 7F,G). DSS increased pro-inflammatory taxa including *Escherichia-Shigella* with LDA score (5.22) Enterobacteriaceae (5.21) *Proteobacteria* (5.23) and Erysipelotrichaceae (4.97). LSP_L favored beneficial genera such as *Bacteroides* (4.69), *Alistipes* (4.16), *Turicibacter* (4.26) and *Ligilactobacillus* (4.97) whereas LSP_H promoted *Rikenella* (4.10), *Odoribacter* (4.07), *Lachnoclostridium* (4.16) and *Marinifilaceae* (4.07). The NC group was dominated by *Muribaculaceae* (5.24), *Parabacteroides* (3.94) and *Alloprevotella* (3.97) reflecting a eubiotic baseline.

At the genus level *Akkermansia* were significantly elevated in DSS mice while LSP treatment particularly high dose effectively restore these taxa (Figures 7H–N). Similarly, *Escherichia-Shigella* expansion in DSS (*p* < 0.0001) was attenuated by both LSP doses. Beneficial genera including *Bifidobacterium, Lactobacillus,* Lachnospiraceae*_NK4A136_group* and *Ruminococcus* were enriched by LSP_H (*p* < 0.001–0.0001) with LSP_L showing intermediate effects illustrating a dose-dependent restoration of favorable microbial populations. Among the beneficial taxa enriched by LSP, *Bifidobacterium* and *Lactobacillus* were most prominent. These genres are likely important mediators of the observed anti-inflammatory and barrier-protective effects.

Family-level analyses corroborate these findings with Lactobacillaceae enriched in LSP_H, *Muribaculaceae* abundant in LSP_L and NC and Enterobacteriaceae expansion prominent in DSS (Supplementary Figure S2). Other families, including Erysipelotrichaceae and *Saccharimonadaceae* showed group-specific patterns consistent with inflammatory dysbiosis.

Collectively these results demonstrate that LSP supplementation particularly at high dose reverses DSS-induced microbial dysbiosis by restoring diversity suppressing inflammatory taxa and enriching beneficial microbial populations. These ecological shifts are likely to contribute to the immunomodulatory and protective effects of LSP observed in previous figures.

### 3.7 LSP restores gut microbial function and ecological balance to support barrier integrity and immune homeostasis

**FIGURE 8:**
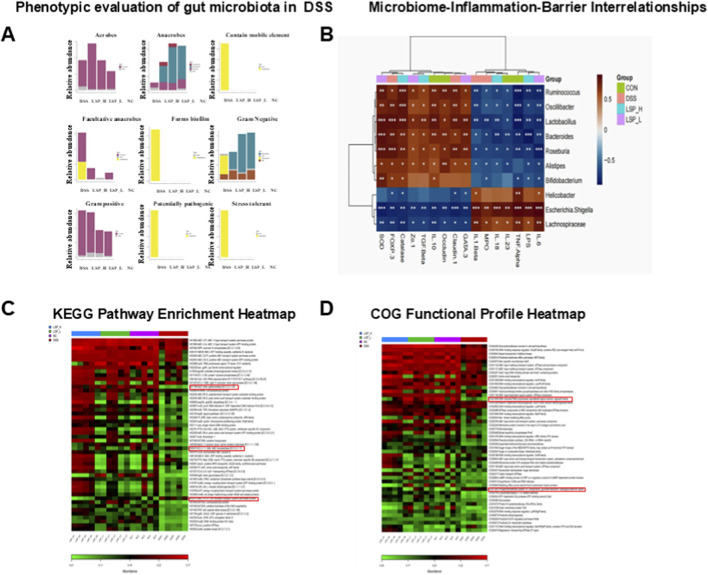
LSP modulates microbial ecology and function in DSS-induced colitis. **(A)** DSS disrupts microbial balance across multiple ecological features; LSP, especially high dose, restores a protective microbiota. **(B)** SCFA-producing and probiotic taxa correlate with anti-inflammatory and barrier-protective markers; pathobionts correlate with inflammation and barrier disruption. **(C)** DSS impairs DNA repair (UvrD/PcrA), which is restored by LSP. **(D)** DSS reduces nitroreductase, glycosyltransferases, and sigma factor, while LSP preserves these functions.

Building on the taxonomic restructuring observed in Figure 7, we next investigated functional and ecological attributes of the gut microbiome to understand how LSP modulates microbial behavior and metabolic potential in colitis (Figures 8A–D).

Ecological profiling revealed pronounced differences across treatments (Figure 8A). LSP_H exhibited the highest abundance of aerobic taxa followed by DSS, LSP_L and NC suggesting that high-dose LSP may favor oxygen-tolerant communities in the recovering gut. DSS samples were enriched in *Firmicutes* whereas LSP_L harbored the highest Bacteroidetes with minor Actinobacteria representation. LSP_H displayed intermediate *Bacteroidetes* abundance and NC samples contained exclusively *Bacteroidetes* reflecting a stable non-inflammatory baseline. Markers of mobile genetic elements indicative of genomic instability were most abundant in DSS consistent with a dysbiotic and potentially pathogenic state. Facultative anaerobes were dominated by *Firmicutes* and *Proteobacteria* in DSS and biofilm-forming taxa were similarly enriched highlighting enhanced adhesion and persistence under inflammatory conditions. Gram-positive taxa mirrored *Firmicutes* distribution, with DSS highest followed by LSP_H, LSP_L, and NC. Notably potentially pathogenic taxa were present only in DSS demonstrating that LSP suppresses harmful microbial populations. Stress-tolerant taxa were also elevated in DSS reflecting adaptation to oxidative and inflammatory pressures (Supplementary Figure S3).

Correlation analysis was performed between the relative abundance of dominant bacterial genera and key inflammatory cytokines, oxidative stress markers, and intestinal barrier–related proteins (Figure 8B). The analysis revealed distinct associations linking microbial composition with host immune and epithelial responses. SCFA-producing genera, including *Ruminococcus*, *Roseburia*, and *Oscillibacter*, showed significant negative correlations with pro-inflammatory cytokines such as TNF-α and IL-23, while exhibiting positive correlations with barrier integrity markers, including ZO-1, Claudin-1, and Occludin, suggesting their protective role in maintaining mucosal homeostasis. In contrast, pathobiont-associated taxa, particularly *Escherichia–Shigella* and *Helicobacter*, were positively correlated with inflammatory mediators (TNF-α, IL-6, and MPO) and negatively correlated with tight junction proteins, indicating their association with intestinal inflammation and epithelial barrier disruption. Moreover, probiotic genera, including *Lactobacillus* and *Bifidobacterium*, were positively associated with anti-inflammatory cytokines and antioxidant indicators, such as IL-10, TGF-β, and SOD, supporting their potential contribution to immune regulation and oxidative balance. Collectively, these correlations suggest that LSP-mediated modulation of gut microbiota is closely linked to reduced inflammation and improved intestinal barrier integrity in DSS-induced colitis.

KEGG Orthology (KO) analysis (Figure 8C) corroborated these observations. DSS exhibited reduced levels of UvrD/PcrA (K00392), a DNA helicase essential for repair and genomic stability. Restoration of UvrD/PcrA and related pathways in LSP-treated groups reinforces their potential role in supporting microbial genomic integrity and promoting host microbe symbiosis during colitis recovery. However, these functional predictions are indirect, and further validation with metagenomic or metabolomic approaches is required to confirm the actual microbial functions.

Functional potential assessed *via* COG annotation (Figure 8D) revealed that DSS significantly downregulated nitro reductase (COG0778), glycosyl transferases involved in cell wall biosynthesis (COG0435, COG0463, COG0551) and the sigma subunit of RNA polymerase (COG1595). These alterations suggest impaired detoxification compromised cell wall integrity and weakened stress response. In contrast NC and LSP-treated groups maintained higher abundances of these functions, indicating preserved microbial structural stability and resilience.

## 4 Discussion

Polysaccharides from edible fungi can modulate gut microbiota and host immunity, yet the structural features governing these effects remain unclear. To narrow this gap, we characterized *Laetiporus sulphureus* polysaccharides (LSP) as a heteropolysaccharide with mixed α- and β-glycosidic linkages and a porous ultrastructure, enabling structure–function association. Its distinct profile compared with *Agrocybe cylindracea* (Atta et al., 2025) and *M. esculenta* (Rehman et al., 2022) indicates potentially unique roles in microbial metabolism, epithelial protection, and immune regulation.

Building on these structural insights, we assessed whether LSP’s unique features translate into protective effects *in vivo*. In DSS-induced colitis, LSP significantly mitigated inflammation As observed in (Kanwal et al., 2020). DSS reduced food and water intake, whereas LSP especially at higher doses restored both. LSP also lowered the disease activity index (DAI), reducing weight loss, bleeding, and diarrhea, and prevented DSS-induced colon shortening. Collectively, these outcomes indicate that LSP effectively targets key pathogenic processes in experimental colitis.

Extending its effects on clinical and colon parameters, histopathology revealed severe mucosal damage and immune infiltration in DSS-treated mice, reflecting epithelial barrier disruption. LSP dose-dependently restored mucosal structure, promoted epithelial repair, and reduced inflammation scores. Consistent with (Li et al., 2024), which reported TSP50 improved crypt morphology and goblet cell numbers, these results indicate LSP preserves epithelial integrity, limits villus loss, and restrains inflammatory infiltration.

Extending its epithelial-protective effects, LSP also mitigated neutrophil-driven oxidative injury in DSS colitis. DSS markedly elevated myeloperoxidase (MPO) activity (Rafeeq et al., 2021), reflecting neutrophil infiltration and tissue damage, whereas LSP dose-dependently suppressed MPO, reduced lipid peroxidation (MDA), replenished GSH, and upregulated T-SOD and CAT. By restoring redox balance, LSP interrupts the cycle of inflammation and oxidative stress (Xu et al., 2024), linking its structural features to colonic protection and highlighting its potential as a microbiota-targeted intervention that modulates both immune and oxidative pathways.

Complementing its anti-inflammatory and antioxidant effects, LSP preserved the mucus–epithelial barrier in DSS colitis. Treatment dose-dependently restored goblet cell density, mucin content (AB and PAS staining), and goblet cell morphology, reinforcing defenses against microbial translocation and dysbiosis (Alioui et al., 2024; Nie et al., 2024).

Mechanistically, LSP’s effects mirror those of golden mushroom (*Cantharellus cibarius* Fr.) compounds, which enhance barrier function and mucin expression (Alioui et al., 2024). By reducing inflammation and oxidative stress while promoting mucosal repair, LSP integrates barrier, immune, and redox homeostasis, highlighting its potential as a microbiota-targeted intervention and the role of polysaccharide structure in barrier protection.

Epithelial barrier integrity depends on both mucus production and tight junctions, which are disrupted in ulcerative colitis. DSS markedly reduced key proteins, including occludin, claudin-1, and ZO-1 (Zhou et al., 2024). LSP dose-dependently restored junctional integrity, with LSP_H strongly upregulating all markers, consistent with studies showing bioactive compounds can counteract DSS-induced tight junction disruption (Zhou et al., 2023).

This coordinated restoration of tight junctions and goblet cell function reinforces mucosal defense against microbial translocation. The pronounced efficacy of LSP_H suggests its structure uniquely strengthens the mucus–junctional axis, surpassing conventional dietary agents, and highlights LSP as a microbiota-targeted therapeutic that enhances both mucosal architecture and barrier function in colitis (Yang et al., 2021).

The mucosal immune environment is central to ulcerative colitis, where dysregulated cytokines drive inflammation and tissue injury. DSS colitis markedly elevates pro-inflammatory mediators such as IL-23, which promotes T cell–driven intestinal inflammation *via* IL-17 and IL-6 signaling (Guo and Lv, 2023; Shao et al., 2024). LSP treatment effectively reduced IL-23, indicating targeted modulation of pathogenic immune pathways and supporting immune homeostasis.

LSP also normalized DSS-induced IL-17 upregulation and restored suppressed anti-inflammatory cytokines IL-4 and IL-10, rebalancing pro- and anti-inflammatory mediators. This dampens Th17-driven responses while promoting a tolerogenic mucosal immune environment, paralleling therapeutic strategies targeting the IL-23/IL-17 axis in disorders like psoriatic arthritis (Sewell and Kaser, 2022), Moreover, LSP reduced TNF-α and IL-6, mirroring effects of raffinose (Raf) (Kim et al., 2023), and mitochondria-targeted antioxidants that alleviate experimental colitis *via* microbiota modulation and inflammasome inhibition (Batista et al., 2020). Similar immunomodulatory effects have been reported for other mushroom polysaccharides, including *G. lucidum* and *L. edodes* which reduce colitis severity through cytokine regulation and enrichment of SCFA-producing microbes. While we did not directly assess NF-κB activation in this study, the structural characterization of LSP revealed a glucose-rich polysaccharide profile consistent with β-glucans and related fungal polysaccharides known to interact with Dectin-1 and TLR2 receptors (Meng et al., 2023; Xie et al., 2019). These receptors are key upstream regulators of innate immune responses and have been shown in other models to modulate NF-κB signaling. NF-κB controls transcription of major inflammatory cytokines, including TNF-α, IL-1β, and IL-6, as well as the anti-inflammatory cytokine IL-10. In our study, DSS exposure increased TNF-α and IL-1β and reduced IL-10, whereas LSP treatment reversed these responses in a dose-dependent manner. Although indirect, this cytokine profile is consistent with potential attenuation of NF-κB–associated inflammatory signaling, providing a plausible mechanism linking LSP composition to immunomodulatory effects.

Mechanistically, LSP tempers innate immune activation and restore mucosal homeostasis by reshaping the cytokine milieu, aligning with multi-target IBD therapies. Whether these effects are direct or microbiota-mediated remains unclear, warranting *in vitro* cytokine and immune-microbiota co-culture studies.

Macrophages are key regulators of intestinal inflammation, with their polarization determining the balance between tissue damage and repair. DSS-induced colitis caused pronounced accumulation of CD68^+^ macrophages in the colonic mucosa, reflecting heightened infiltration and activation, consistent with previous reports (Ramos et al., 2023). Quantitative analysis confirmed that DSS increased CD68 density, which was significantly reduced by both low- and high-dose LSP, paralleling prior interventions that modulate macrophage recruitment to restore mucosal homeostasis (Xiao et al., 2024).

Beyond recruitment, macrophage polarization critically influences disease progression. DSS elevated the pro-inflammatory M1 marker CD86, whereas LSP suppressed CD86 and restored the anti-inflammatory M2 marker CD163. This shift toward M2 macrophages indicates that LSP reprograms the mucosal immune environment, mitigating inflammation and promoting repair (Xiong et al., 2023). The precise mechanisms direct or indirect remain unclear, warranting future *in vitro* studies with macrophage or T cell assays and receptor-targeted experiments.

These findings show that LSP limits macrophage infiltration and orchestrates their functional phenotype, coupling innate immune modulation with barrier and cytokine restoration. By promoting an M2-dominant, anti-inflammatory milieu, LSP emerges as a multifaceted agent harmonizing immune regulation and epithelial protection. T cell transcription factors further shape mucosal immunity. In DSS-treated mice, GATA-3 was reduced, contrasting reports linking its overexpression to worsened colitis *via* IL-13 (Wan et al., 2024) highlighting context dependent effects. LSP modestly restored FOXP-3, consistent with the role of regulatory T cells (Tregs) in suppressing colitis and maintaining homeostasis (Sharabi and Tsokos, 2020).

Together, these observations suggest that while GATA-3 effects vary, LSP’s promotion of FOXP-3^+^ Tregs provides a robust mechanism to mitigate inflammation, complementing its modulation of macrophages, cytokines, and barrier function to rebalance the intestinal immune landscape.

Gut microbial diversity is essential for intestinal homeostasis and is disrupted in DSS-induced colitis. LSP supplementation dose-dependently restored Chao1 and Shannon indices, reflecting recovery of richness and evenness, consistent with reports on *Meconopsis* (Zheng et al., 2024), *Banxia Xiexin decoction* (Luo et al., 2024), and *adzuki* bean seed coat (Yin et al., 2025). Other bioactive, including Dihydromyricetin (DMY) and Hylocereus undatus flower extract (HUF), similarly restore microbial balance and support barrier integrity (Wang et al., 2025; Liao et al., 2023).

Beta-diversity analyses showed DSS samples clustered separately from controls across PCA, PCoA, and NMDS (Figures 5C–E), indicating major compositional shifts. LSP_H repositioned communities closer to controls, suggesting near-complete restoration, paralleling interventions like Arbutin with DSS (Qin et al., 2024) and HUF supplementation (Li et al., 2025).

LefSe analysis showed that DSS selectively enriched proinflammatory taxa, including *Escherichia-Shigella*, Enterobacteriaceae, *Proteobacteria*, and Erysipelotrichaceae, consistent with their known roles in DSS-induced colitis (Wu et al., 2023). *Proteobacteria* exacerbate inflammation, while *Escherichia-Shigella* drive proinflammatory responses. For instance, *Akkermansia* was elevated in DSS mice, which under inflammatory stress may exacerbate mucin degradation, but under balanced conditions can support SCFA production and regulatory T cell responses (Miao et al., 2024; Qu et al., 2023). In contrast, LSP promoted beneficial taxa such as *Bacteroides*, *Alistipes*, *Turicibacter*, and *Ligilactobacillus*, which support barrier function and produce anti-inflammatory short-chain fatty acids (Ma et al., 2025). Healthy controls were dominated by *Muribaculaceae*, *Parabacteroides*, and *Alloprevotella*, recognized core commensals with probiotic or anti-inflammatory potential (Dai et al., 2025).

LSP_H further increased *Rikenella*, *Odoribacter*, *Lachnoclostridium*, and *Marinifilaceae*. While less studied, *Odoribacter* and *Lachnoclostridium* are butyrate producers, *Rikenella* has context-dependent effects, and *Marinifilaceae* are poorly characterized (Tang et al., 2023). Overall, LSP reversed DSS-induced dysbiosis by suppressing proinflammatory taxa, restoring *Lactobacillus*, and enriching SCFA-associated microbes. LSP_H additionally promoted *Bifidobacterium* and other potentially beneficial taxa, reflecting broader microbial restructuring. *Lactobacillus* recovery aligns with its anti-inflammatory role (Shi et al., 2021), while *Bifidobacterium* modulation appears context-dependent, suggesting nuanced effects. *Bifidobacterium* and *Lactobacillus* likely play central roles in LSP-mediated protection, warranting future validation *via* gnotobiotic models or *in vitro* co-cultures to confirm their contributions to immune modulation and barrier integrity.

Functional profiling showed that DSS suppressed key microbial functions, including nitroreductase, glycosyltransferases, and the RNA polymerase sigma subunit, indicating impaired detoxification, cell wall integrity, and stress responses. LSP preserved these functions, aligning with reports that dysbiosis disrupts core microbial capabilities (Lv et al., 2025). KEGG analysis further revealed DSS-induced reduction of UvrD/PcrA, a DNA helicase essential for genomic stability, which was restored by LSP, suggesting protection of microbial DNA repair mechanisms (Sun et al., 2020). These inferences, however, are based on 16S rRNA profiles and may not reflect actual gene expression or enzymatic activity; metagenomic, meta transcriptomic, or metabolomic studies are needed to confirm these functional shifts and their impact on host physiology.

Correlation analyses supported these findings: SCFA-producing genera like *Roseburia* and *Ruminococcus* were negatively associated with proinflammatory cytokines and positively with tight junction markers, reinforcing their role in barrier integrity (Yang et al., 2022). In contrast, pathogenic taxa such as *Escherichia*, *Shigella*, and *Helicobacter* correlated positively with inflammatory markers (Hu et al., 2022). Beneficial taxa including *Lactobacillus*, *Bifidobacterium*, and Lachnospiraceae*_NK4A136_group* were linked to anti-inflammatory cytokines, reflecting immunomodulatory potential (de Oliveira et al., 2025), while *Alistipes* showed negative associations with oxidative stress markers, consistent with SCFA-mediated antioxidant activity (Paolillo et al., 2020). Together, these results indicate that LSP not only reshapes microbial composition but also preserves functional capacities critical for gut homeostasis and mucosal protection.

This study has limitations: LSP structural analysis was limited to basic characterization without advanced techniques (e.g., NMR, linkage analysis), and the high doses used in mice may not directly translate to humans. Microbial functional predictions relied on 16S rRNA inference rather than metagenomics or metabolomics, and the mechanisms of LSP’s immune modulation direct receptor interaction *versus* microbiota-derived metabolites remain unclear. Nonetheless, this is the first study demonstrating that LSP mitigates colitis by reshaping microbial ecology and rebalancing cytokines. Strengths include integrating histological, immunological, and microbial data for a holistic view of host-microbe interactions. Future studies should employ detailed structural analyses, optimized dosing, metabolomic validation, and mechanistic experiments in gnotobiotic or immune-cell models to enhance translational relevance.

## 5 Conclusion

This study demonstrates that crude polysaccharides from *L. sulphureus* (LSP) exert protective effects against DSS-induced colitis by restoring intestinal homeostasis. LSP reinforced mucosal integrity, rebalanced immune responses, alleviated oxidative stress, and remodeled the gut microbiota toward beneficial, SCFA-producing taxa. Together, these findings position LSP as a promising multi-targeted natural candidate for gut health. While further work is required to define its active structural motifs, optimize dosing, and validate mechanisms in advanced models, our results provide a mechanistic foundation for future preclinical development of LSP in inflammatory bowel disease.

## Data Availability

The original contributions presented in the study are publicly available. The 16S rRNA sequencing data have been deposited in the NCBI Sequence Read Archive (SRA) under BioProject accession number PRJNA1446982.
