# Correction: *Laetiporus sulphureus* polysaccharides mitigate colitis by reshaping the gut microbiota and regulating immune responses

**DOI:** 10.3389/fphar.2026.1905872

**Published:** 2026-07-09

**Authors:** Sharafat Ali, Yamina Alioui, Imran Khan, Hidayat Ullah, Mujeeb Ur Rahman, Aamna Atta, Mohammed Abusidu, Muhammad Ilyas, Uzma Noor, Renzhen Ma, Muhsin Ali, Nabeel Ahmed Farooqui, Ting Deng, Guangyang Wang, Yi Xin, Shanshan Sha, Yufang Ma

**Affiliations:** 1 Department of Biochemistry and Molecular Biology, College of Basic Medical Science, Dalian Medical University, Dalian, China; 2 Department of Biotechnology, College of Basic Medical Science, Dalian Medical University, Dalian, China; 3 Department of Microecology, College of Basic Medical Science, Dalian Medical University, Dalian, China; 4 Guangdong Provincial Key Laboratory of Research and Development of Natural Drugs, and School of Pharmacy, Guangdong Medical University, Dongguan, China; 5 Department of Physiology, College of Basic Medical Sciences, Dalian Medical University, Dalian, China

**Keywords:** epithelial barrier, gut microbiota, immune modulation, *laetiporus sulphureus* polysaccharides, ulcerative colitis

In the published article, there was an error in Figure 6C as published. The column headers “Merge” and “DAPI” were inadvertently transposed.

**FIGURE 6:**
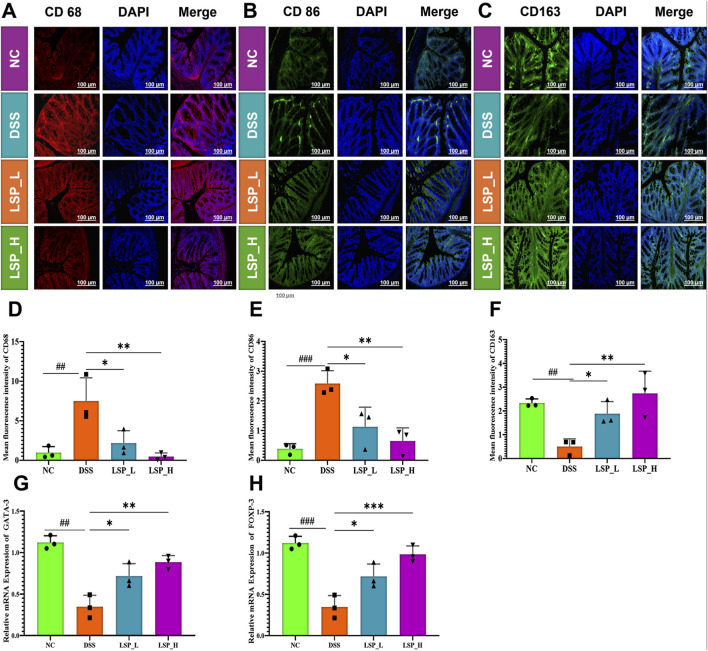
LSP modulates immune cell programs in DSS-induced colitis. **(A–C)** IF images of CD68, CD86, CD163 macrophages; **(D–F)** quantification showing DSS-induced macrophage infiltration and reversed by LSP; **(G,H)** GATA-3 and FOXP-3 expression in Th2 and Treg cells restored by LSP. All data were presented as mean ± SD. Statistical analysis was performed using one-way ANOVA followed by Tukey’s post hoc test. *p < 0.05, **p < 0.01, ***p < 0.001.

The labels were published as: “CD163 | Merge | DAPI” The correct order is: “CD163 | DAPI | Merge”

This error concerns only the figure panel labels. The fluorescence images, underlying experimental data, results, interpretation, and conclusions of the study remain unchanged.

The corrected **Figure 6** and its caption appear below.

The original article has been updated.

