# Supplementary material for: Laetiporus sulphureus polysaccharides mitigate colitis by reshaping the gut microbiota and regulating immune responses
Source: Front Pharmacol. 2026 Apr 15;17:1705032. doi: 10.3389/fphar.2026.1705032 (PMC13125129; doi:10.3389/fphar.2026.1705032)
Supplement: Supplementary file 1 [file Table1.docx]

| **Gene** | **Forward Primer (5'->3')** | **Reverse Primer (5'->3')** |  |
| --- | --- | --- | --- |
| FOXP-3 | CAGCTGCCTACAGTGCCCCTAG | CATTTGCCAGCAGTGGGTAG | |
| GATA-3 | GAACCGGCCCCTCATTAAG | ACATATCTTCACGCATGCGG | |
| IL-4 | ACAGGAGAAGGGACGCCAT | GAAGCCCTACAGACGAGCTCA | |
| IL-23 | TGTGCTCCTCTTGAAGATGTGCC | CTGGAGGAGTTGGCTGAGTAACA | |
| IL-1β | GCAACTGTTCCTGAACTCAACT | ATCTTTTGGGGTCCGTCAACT | |
| IL-10 | CCAAGCCTTATCGGAAATGA | TTTTCACAGGGGAGAAATCG | |
| TNF-α | CATCTTCTCAAAATTCGAGTGACAA | TGGGAGTAGACAAGGTACAACCC | |
| IL-6 | CCACTTCACAAGTCGGAGGCTTA | GCAAGTGCATCATCGTTGTTCATAC | |
| TGF-β | CTCCCGTGGCTTCTAGTGC | GCCTTAGTTTGGACAGGATCTG | |
| IL-17 | TTTAACTCCCTTGGCGCAAAA | CTTTCCCTCCGCATTGACAC | |
| β-actin | GGCTGTATTCCCCTCCATCG | CCAGTTGGTAACAATGCCATGT | |

**Table 1.** List of Primers used.
