# Supplementary material for: Laetiporus sulphureus polysaccharides mitigate colitis by reshaping the gut microbiota and regulating immune responses
Source: Front Pharmacol. 2026 Apr 15;17:1705032. doi: 10.3389/fphar.2026.1705032 (PMC13125129; doi:10.3389/fphar.2026.1705032)
Supplement: Supplementary file 2 [file Presentation1.pptx]

## Slide 1
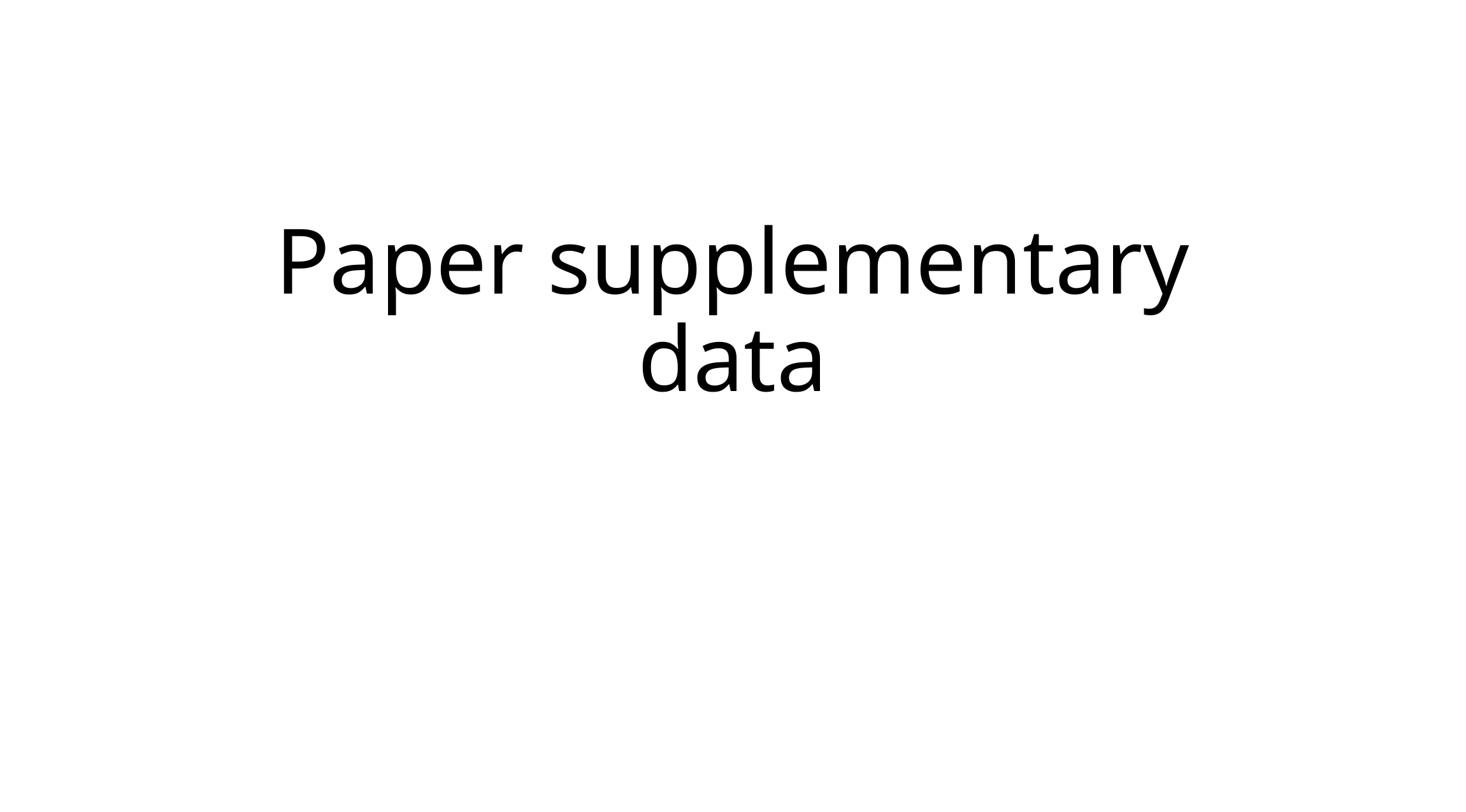

# Paper supplementary data

## Slide 2
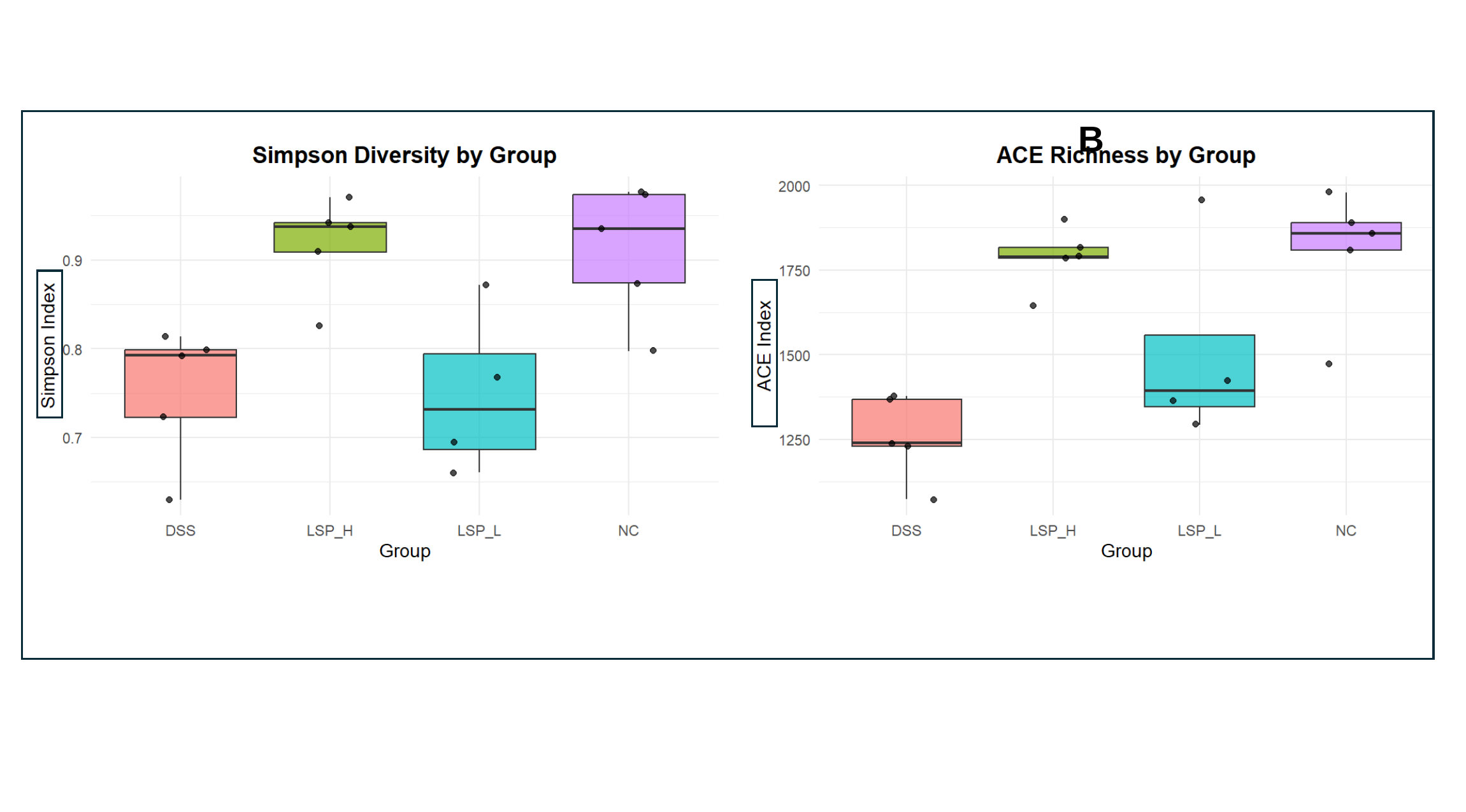

A						 								 B

## Slide 3
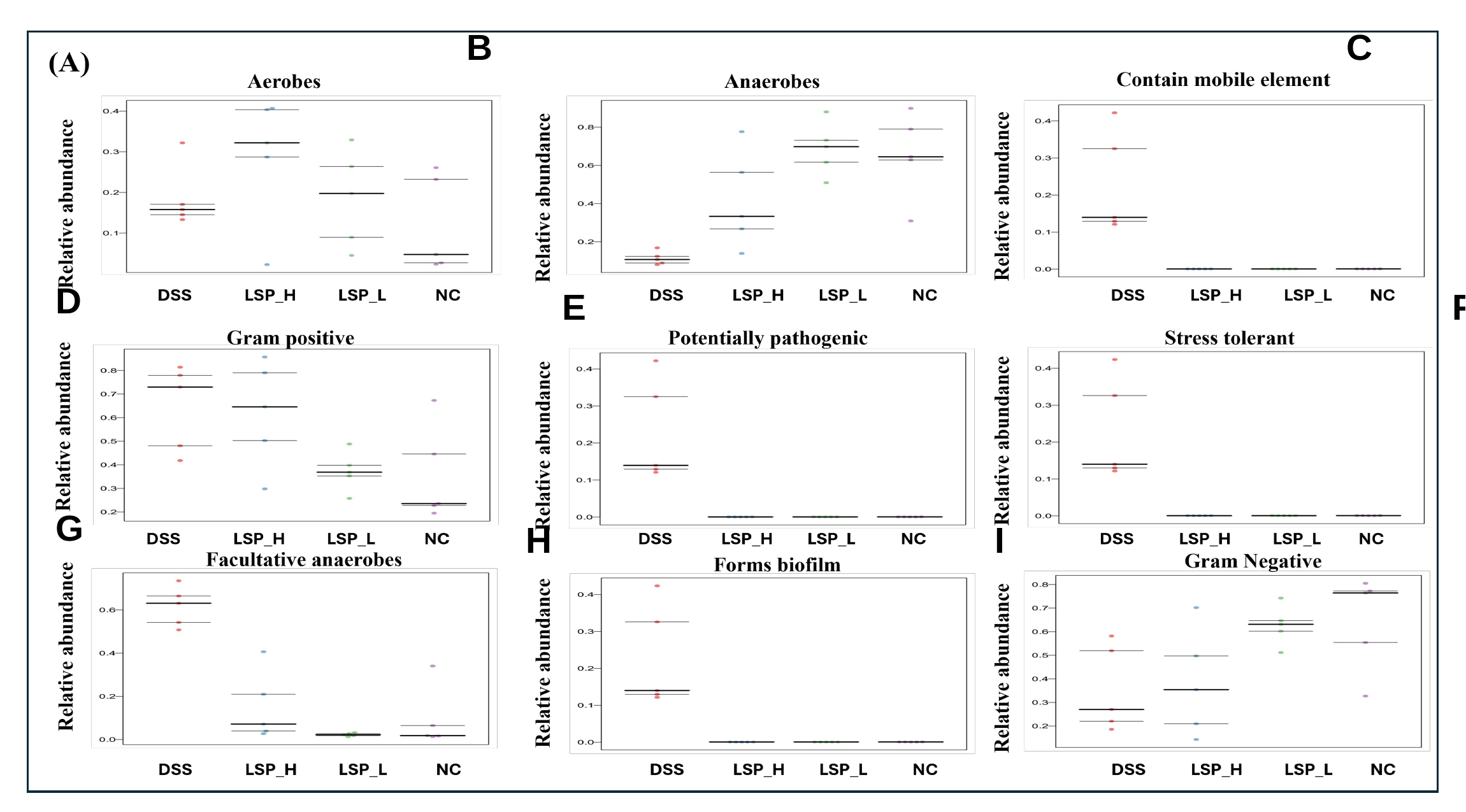

A						 			B									 C
										E									 F
D
G
H
I

## Slide 4
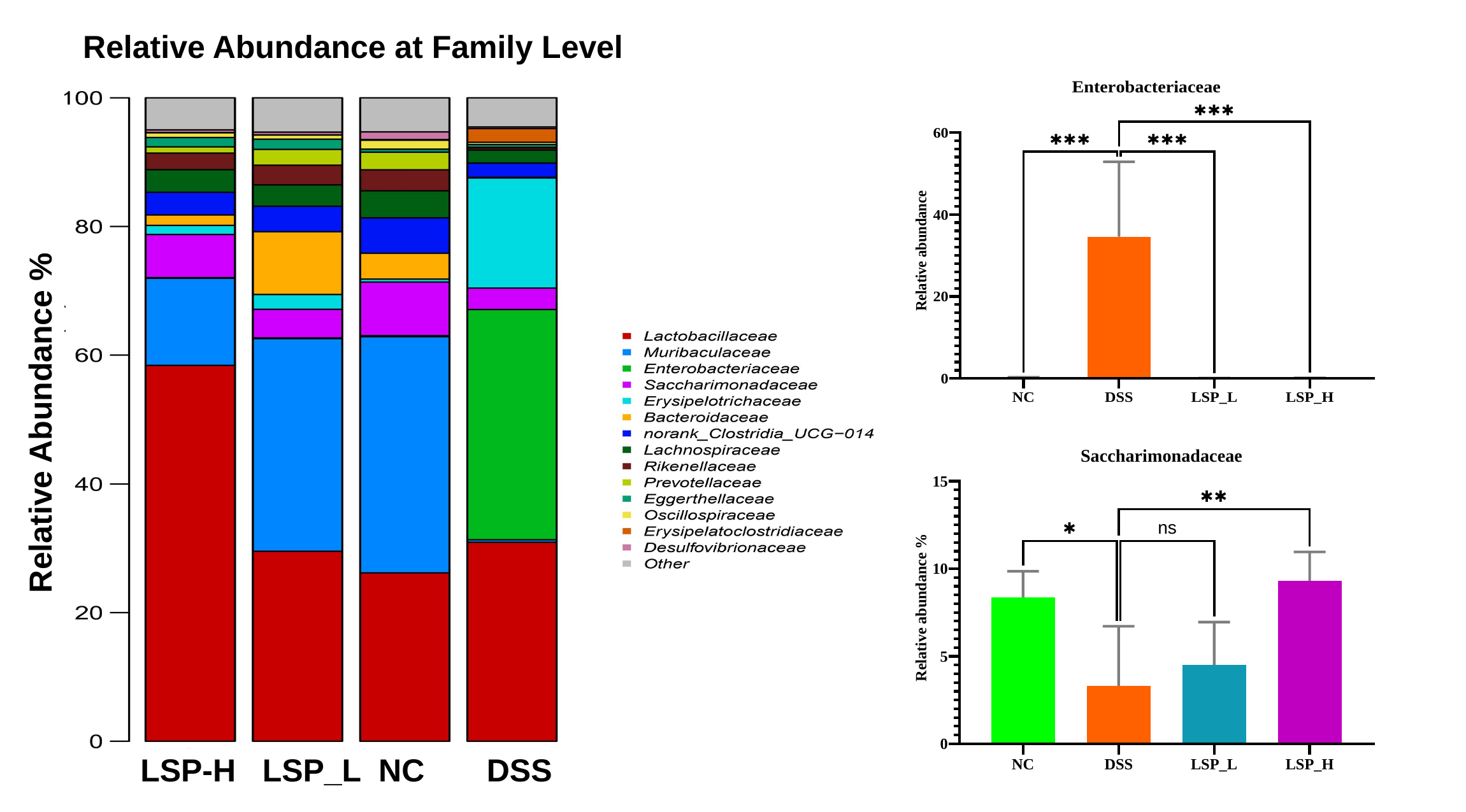

Relative Abundance at Family Level
Relative Abundance %
LSP-H LSP_L NC DSS
